# Does extreme asymmetric dominance promote hybridization between *Anopheles coluzzii* and *Anopheles gambiae* s.s. in seasonal malaria mosquito communities of West Africa?

**DOI:** 10.1186/s13071-015-1190-x

**Published:** 2015-11-11

**Authors:** Abdoulaye Niang, Patric S. Epopa, Simon P. Sawadogo, Hamidou Maïga, Lassana Konaté, Ousmane Faye, Roch K. Dabiré, Frédéric Tripet, Abdoulaye Diabaté

**Affiliations:** Institut de Recherche en Sciences de la Santé, Bobo-Dioulasso, Burkina Faso; Centre for Applied Entomology and Parasitology, School of Life Sciences, Keele University, Staffordshire, UK; Laboratoire d’Ecologie Vectorielle et Parasitaire, Faculté des Sciences et Techniques, Université Cheikh Anta Diop, Dakar, Senegal

## Abstract

**Background:**

*Anopheles gambiae* s.s. and *An. coluzzii* are two of the most important malaria vector species in sub-Saharan Africa. These recently-diverged sibling species do not exhibit intrinsic post-zygotic barriers to reproduction and are thought to be separated by strong assortative mating combined with selection against hybrids. At present, little is known about the ecological conditions that determine hybridization and introgression between these cryptic taxa.

**Methods:**

Swarm segregation and assortative mating were studied in Western Burkina Faso in the villages of Vallée du Kou (VK7) and Soumousso which differed in terms of which sibling species was much rarer than the other, and in Bana where both occurred in similar proportions. Swarms and pairs in copula were collected and genotyped, the proportion of intra and interspecific mating determined, and interspecific sperm transfer checked genetically. Females were collected through larval and adult indoor collections and genotyped or sexed-and-genotyped via a novel multiplex PCR.

**Results:**

A total of 3,687 males and 220 females were collected and genotyped from 109 swarms. Only 3 swarms were composed of males from both species, and these were from the village of VK7 where *An. gambiae* s.s. was comparatively rare. Mixed-species pairs captured in copula were only detected in that area and made for 3.62 % and 100 % of mating pairs involving *An. coluzzii* and *An. gambiae* s.s. individuals, respectively. The high *An. gambiae* s.s. cross-mating rate was mirrored by high rates of hybridizations estimated from female larvae and adults indoor collections. This contrasted with Soumousso, where despite being much less common than *An. gambiae* s.s., *An. coluzzii* males did not form mixed swarms, females were not found in interspecific swarms or copula and hybridization rates were low in both sibling species.

**Conclusions:**

These data suggest that ecological conditions leading to rare *An. gambiae* s.s. in populations dominated by *An. coluzzii* may promote a breakdown of spatial swarm segregation and assortative mating between the two species. The lower overall hybridization rates observed at the larval and adult indoor stages compared to cross-mating rates support the idea that post-mating selection processes acting against hybrids may occur mostly prior to and/or at the early larval stages.

## Background

The *Anopheles gambiae* complex is responsible for the vast majority of malaria transmission in sub-Saharan Africa [1, 2]. Within the complex, the sibling species *Anopheles coluzzii* Coetzee & Wilkerson and An. *gambiae s.s*. Giles, formerly known as the M and S molecular form of *An. gambiae s.s*. [3], are dominant in many malaria vector communities [1, 4]. These cryptic taxa are thought to be undergoing a process of speciation with gene flow [1, 5, 6] and understanding their mechanisms of pre-mating and post-mating reproductive isolation has important implications for vector control [7, 8].

In the laboratory, hybridization between the sibling species produces fertile and viable 1st generation progeny and backcrosses [9]. Therefore, reproductive isolation in natural populations is thought to occur through strong assortative mating combined with selection against hybrids [10, 11]. Of these two processes, the first is the best documented [8]. The two species mate in flying aggregations formed by males at dusk called swarms. These swarms are visited by females to find a mate, leading to the formation of a mating pair that leaves the swarm in copula [8, 12]. Assortative mating occurs via a combination of spatial swarm segregation [13, 14] and, within swarms, by short-range recognition mechanisms, among which flight-tones are believed to play an important role [8, 15]. In most parts of West Africa, despite these strong pre-mating barriers, cross-insemination between the two species occurs occasionally, resulting in rare hybrids [5, 10]. One crucial consequence of such residual gene flow for vector control is that pesticide resistance genes can introgress between the two species, as recently described in Burkina Faso and Mali [16–19]. Another consequence is that genetic vector control programmes aiming to spread genes of refractoriness to *Plasmodium* across populations could potentially take advantage of this residual gene flow to impact several target populations simultaneously [8]. There are however exceptions to this pattern of limited local hybridization. Along the Western coast of Africa, several hybrid zones have been observed where gene flow is thought to be much higher, [20, 21] suggesting either a different mating behaviour or looser selection pressure against hybrids [22–24]. Whether these hybrid zones are a recent phenomenon due to environmental changes as suggested by some authors [3] or have been in place for much longer is currently unknown.

Divergent ecological adaptations between the sibling species have been shown for a variety of traits, including larval anti-predator responses, larval habitat segregation, and aestivation strategies [25–28]. However, the prediction that these differences should result in decreased fitness in *F*_1_ hybrids (1st generation hybrids) and *F*_1+n_ hybrids (*F*_2_ backcrosses and recombinants from subsequent generation) is currently supported by population genomic data rather than from direct field experimental evidence. Comparative genomic studies have shown that *An. coluzzii* and *An. gambiae* s.s. populations are consistently genetically differentiated at pericentromeric regions of the X, 2 L and 3 L chromosomes [29, 30]. These so-called ‘islands of speciation’ are thought to be key to assortative mating as well as ecological divergence [6]. Whether genetically differentiated areas detected outside the islands in some studies are the result of population-level differential selection to specific habitats or reflect genome-wide patterns differentiation associated with a more advanced stage of speciation of the sibling species is still debated [5, 31, 32]. Crucially, the pericentromeric islands typical of each subtaxa were found to be consistently genetically associated amongst populations, even those undergoing hybridization, supporting the notion that *F*_1+n_ hybrids at those loci are selected against in natural populations [23, 24, 30]. More recently, sympatric populations in Ghana, Burkina Faso and Mali have recently witnessed the introgression of the entire 2 L pericentric island of *An. gambiae* s.s. containing pesticide resistance loci, into *An. coluzzii* [19, 32]. Interestingly, following the initial genetic sweep, *An. coluzzii* populations have been observed to regain their original *An. coluzzii*-like pericentric 2 L region thereby further strengthening the hypothesis of selection against hybrids [19].

Comparative genomics analyses also suggest that introgression between *An. coluzzii* and *An. gambiae* s.s. is asymmetrical and occurs more commonly from the former to the later [23]. In this regard, the recent adaptive introgression of the 2 L pericentric island from *An. gambiae* s.s. into *An. coluzzii* is proof that gene flow can sometimes occurs in the opposite direction [19, 32]. Population genomic studies also hypothesized that *An. gambiae* s.s. females may be more prone to mating with heterospecific males than *An. coluzzii* females [5]. Male anophelines are hemizygous at the X chromosome inherited from their mother. In the first large study of the spatiotemporal dynamics of hybridization between the sibling species, 18 out of 19 *F*_1_ hybrid males collected in Guinea Bissau possessed an *An. gambiae* s.s. maternal X chromosome [5]. Thus, baring the possibility of strong selection acting specifically against hybrid male progeny from *An. coluzzii* females, this suggests that *An. gambiae* s.s. females are more permissive to cross-mating [5].

At present very little is known of the ecological conditions that lead to the spatial and temporal patterns of gene flow reported in West Africa. One possible explanation would be that the extreme seasonal variation in abundance of the sibling species observed in many sites of the Soudanian and Sahelian areas could interfere with key processes of assortative mating. *Anopheles coluzzii* and *An. gambiae s.s.* populations in these ecological zones differ in their draught tolerance and aestivation strategies [28, 33, 34]. Whilst *An. coluzzii* populations reduce their reproduction during the dry season and aestivate at low densities locally, *An. gambiae* s.s. populations crash towards the end of the wet season and disappear completely during the dry season [34]. It is unknown whether *An. gambiae* s.s. individuals aestivate in neighbouring, more clement regions where breeding might continue. However, studies have shown that in the subsequent wet season *An. gambiae* s.s. reappears in sympatric populations long after the resurgence of *An. coluzzii* [34, 35]. Depending on local environmental conditions such as ambient humidity and rainfall, *An. gambiae* s.s. may outcompete *An. coluzzii* and become the dominant species or remain the minor one.

The onset of the rainy season that sees *An. coluzzii* being extremely dominant in Soudanian and Sahelian malaria vector communities [34, 35] could be crucial to the incomplete and asymmetric reproductive isolation observed between these two species. Recent studies conducted on spatial swarm segregation between *An. coluzzii* and *An. gambiae* s.s. in Western Burkina Faso give insight into how this might affect swarm dynamics. The analyses of a large number of swarms revealed a strong correlation between the number of males swarming and the number of mating pairs and unpaired females captured in swarms [36, 37]. When one species is very dominant leading to a high density of large swarms, males of the minor species may have difficulties in finding conspecific males and forming and maintaining monospecific swarms [36, 37]. This may explain the higher occurrence of mixed-species swarms reported in such environment [37, 38]. Furthermore, if females cannot find conspecific swarms easily due to their rarity or limited size and attractiveness, they may have to settle for more conspicuous interspecific swarms and mate disassortatively. If this hypothesis is true, other ecological conditions that result in strong dominance of one population over the other could potentially result in disruption of assortative mating. Intensive rice cultivation, for example, creates an abundance of larval breeding sites that better suit *An. coluzzii* larvae and promote its strong dominance throughout the year.

These ecological setting could also explain the apparent asymmetry in cross-mating permissiveness between the two species suggested by the genomic evidence as these studies stem from the Soudanian and Sahelian ecological zones. Field studies have examined evidence for interspecific mating amongst the two sibling species through the detection of mixed-species copula in swarms and/or detection of heterospecific males in their spermatheca are rare given the amount of time they require either for sampling pairs in copula from swarms and/or to conduct sperm analyses. In the first paper describing the strength of assortative mating between the two species in sympatric populations in Mali, 1 of 55 (1.82 %) *An. gambiae* s.s. and 2 of 195 (1.03 %) of *An. coluzzii* females were cross-inseminated [10]. A recent study of swarms conducted in two villages of Burkina Faso, where mosquito communities were dominated either by *An. coluzzii* or *An. gambiae* s.s., identified 4 interspecific copulae out of a total of 143, 3 of which involved *An gambiae* s.s. females [38]. Therefore, the data suggest that *An. gambiae s.s* females might be more prone to cross-mating than those of *An. coluzzii*, but is currently too scant to statistically support this hypothesis.

In the following study, extensive swarm sampling was again conducted in Western Burkina Faso in the villages of Vallée du Kou (VK7) and Soumousso where one sibling species was each time much rarer than the other, as well as in the village of Bana where the two occurred in similar proportions. A large sampling and genotyping effort was undertaken to detect mixed-males swarms, females visiting heterospecific swarms, and intra and interspecific mating pairs. Finally, the sperm of females found in copula with heterospecific males was genetically analyzed using a DNA extraction protocol for small amounts of starting material. The resulting mixed-mating pairs rates were compared to hybridization rates measured from female larvae that were sexed molecularly and genotyped using a single multiplex Polymerase Chain Reaction (PCR) [39, 40]. They were also compared to hybridization rates calculated from indoor resting female catches. In addition to clarifying the putative role of extreme species dominance in asymmetric disruption of reproductive isolation, the comparison of hybridization rates at different life stages offer some insights on possible sources of selection pressure against hybrids. These findings are relevant to our understanding of gene flow patterns amongst population of African malaria vectors with important implications for their vector control.

## Methods

### Study sites

Vallée du Kou is a rice-growing area developed in the early 1970’s in Western Burkina Faso, about 30 km North of Bobo-Dioulasso and situated between 4° 24’ 42“W longitude and 11° 23′ 14”N latitude. It contains seven villages totalling 4470 habitants. The survey was conducted in VK7, located on the boundary of the rice field and cotton cultivation area characterized by wooded savannah. The mean annual rainfall is about 1200 mm and the area is characterized by a rainy season from May to October and a dry season from November to April. The river Kou is a permanent source of irrigation and there are 2 rice crops per year (January-May and July-November). Because of the irrigation system, the rice fields form permanent mosquito breeding sites that are preferentially colonized by *An. coluzzii*. During the rainy season, additional rain-dependent breeding sites are very often found in depressions and ponds, allowing the development of *An. gambiae* s.s..

Soumousso (11°00’46“N, 4°02′45”W) and Bana (12°36′00“N, 3°28′59”W) are villages located in the humid savannah of Western Burkina Faso. As in VK7 the rainy season occurs from May to October followed by a long dry season from November to April. The average annual rainfall ranges from 1000–1200 mm. In Soumousso, both incipient species coexist in sympatry and their highest density occurs in September. However, the relative frequencies of the two species change over time with *An. gambiae* s.s. being predominant from July to November, and *An. coluzzii* dominating from December to June. *Anopheles funestus, An. arabiensis* and *An. nili* are also found at lower densities. The village of Bana is characterized by comparable seasonality but has more balanced densities of *An. coluzzii* and *An. gambiae* s.s.*.*

### Swarm monitoring

An exhaustive survey of swarms was undertaken by trained observers in the three study sites: VK7, Soumousso and Bana from July to November 2011. Swarm locations were mapped using a global positioning system (GPS) GARMIN, series GPSMAP®62.2.3 with measurements of latitude and longitude accurate to within 3 m. Observations were made to identify swarm sites scattered throughout the entire villages that were used every evening. All swarms were then sampled 6 times every month from July to November using insect nets as described previously [13, 14, 41]. In VK7 and Soumousso, mating pairs were also collected in order to estimate the rate of intraspecific versus interspecific mating. To ensure equal sampling of all swarms, two capturers attended the known swarming sites and spent the first 15 min from the initiation of the mating swarm (first male arriving) capturing mating pairs before they collected the whole swarm. Mating pairs were collected as they formed and fell or flew out of swarms. Mosquitoes were aspirated into separate cups for each swarms, killed with ethylic ether, identified morphologically as *An. gambiae* s.l. [42] and kept in 70 % ethanol in 1.5 ml tubes. All sampled males, females from mating pairs, and unpaired females were then again checked morphologically before being genotyped to species level (see below).

### Adult females and larvae sampling

During the same period as swarm monitoring, indoor resting females of *An. gambiae* s.l. were collected from houses or inhabited huts from the villages of Soumousso and VK7 using mouth aspirators. They were described morphologically as described above and stored in 70 % ethanol before being genotyped to species level (see below).

*Anopheles gambiae* s.l larvae were collected from several pools of water spread throughout the villages of Soumousso and VK7 on the third week of each month to ensure that larval and adult cohorts were collected at similar periods in each village. All collected mosquito larvae were examined carefully to separate anopheline and culicine larvae before classifying them from 1^st^ to 4^th^ instars and immediately preserving them in 70 % ethanol for subsequent DNA analysis. The larval site types including footprints, puddle, pond, and rice field were recorded.

### Mosquito identification by Polymerase Chain Reaction (PCR)

DNA was extracted from males and females from swarms and indoor residual fauna that were identified morphologically as *An. gambiae* s.l. [42]. A single leg or other part of the carcass was used excluding female abdomens to avoid possible contamination with DNA from sperm in their spermathecae. Individual DNA extractions were genotyped to species level by PCR [40] and putative hybrids further confirmed by PCR followed by Restriction Fragment Length Polymorphism genotyping as described in [43].

Females caught in copula with a heterospecific male were dissected in order to confirm effective cross-insemination. Their spermatheca was isolated, broken open, and the sperm bundle transferred to a 1.5 ml centrifuge tube as described in previous studies [10, 44]. DNA extractions were done using the ChargeSwitch® gDNA Micro Tissue Kit (Life Technologies, USA) following the manufacturer’s instructions. The sperm DNA was then analyzed using the diagnostic PCR-RFLP [43].

**Fig. 1:**
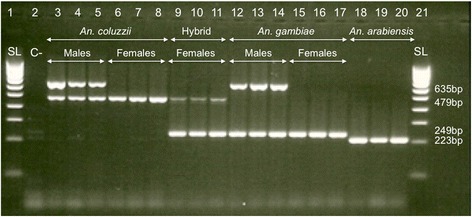
Multiplex PCR for simultaneous sexing and species identification of *An. gambiae* s.l. larvae. The primers developed for a species identification diagnostic [40] and for amplification of a male Y-chromosome specific sequence [39] were combined in a single optimized PCR reaction. The lanes were: DNA size ladder (SL, 1 and 21); negative control (C-, 2); males (3–5) and females (6–8) *An. coluzzii* bands, hybrid female bands (9–11), males (12–14) and female *An. gambiae* s.s. (15–17) bands and *An. arabiensis* (18–20) female bands. Expected sizes are indicated in base-pairs (bp)

DNA was extracted from individual larvae using a standard protocol [38]. Because hybrid males have a single copy of the hemizygous X chromosome, they cannot be identified using classic molecular diagnostics based on polymorphisms in the rDNA region on that chromosome. Consequently, all larvae were sexed and genotyped to the species level using a single multiplex PCR that combined the Y-chromosome specific primers used for sperm detection [39] with primers targeting a specific insertion of a SINE (Short Interspersed Element) located in division 6 of the X-chromosome and commonly used for species identification [40]. The primers (S200X6-1 F 5′-TCGCCTTAGACCTTGCGTTA-3′; S200X6-1R 5′-CGCTTCAAGAATTCGAGATAC-3′ for the SINE diagnostic, and S23-F 5′-CAAAACGACAGCAGTTCC-3′; S23-R 5′-TAAACCAAGTCCGTCGCT-3′) for Y-chromosome detection, were combined into a single reaction after checking for possible dimer problems using the tool available at http://www.thermoscientificbio.com/webtools/multipleprimer/. A PCR mixture contained 2 μl template DNA, 1.5 mM MgCl_2_, 0.5 μl of 5x buffer, 0.2 mM of each dNTPs, 1.5 pmol of each S200X6.1 primers, 2 pmol of each S23 primers and 0.05 units of Taq polymerase for a total volume of 25 μl was used. The PCR reactions were performed on a Bio-Rad S1000™ thermocycler with an initial denaturation at 94 °C for 10 min, followed by 35 cycles of 94 °C for 30 s, 54 °C for 30 s, and 72 °C for 1 min, followed by a final elongation step at 72 °C for 10 min. The PCR products were separated by gel electrophoresis to generate diagnostic sex and species-specific banding phenotypes (Fig. 1). Male samples from larval cohorts and their genotypes were not used in further analyses.

### Statistical analyses

Differences in hybridization rates at the mating stage (rates of mixed-species to interspecific pairs), the female larval and adult stages (rates of hybrids to non-hybrid females) in the VK7 and Soumousso populations, as well as possible selection acting against hybrids from the mating to larval and to adult stage were analyzed using Logistic Regression modelling. The probabilities associated with main effects are Chi-square likelihood-ratio tests and pair-wise comparisons of different levels within effects (e.g. mating, adult and larval stage) are Chi-square likelihood-ratio tests conducted on the odd-ratios generated by the Logistic Regression. Critical pair-wise comparisons were further confirmed using Fisher-Irwin exact tests. Both maximum-likelihood and exact test approaches are typically more robust to low cell counts than the Pearson Chi-square tests [45]. All statistical analyses were carried out using JMP (SAS Institute inc.).

## Results

### Swarm sampling and analyses

A total of 109 swarms were sampled for mating pairs and collected from July to November 2011 in the 3 study sites.

**Fig. 2:**
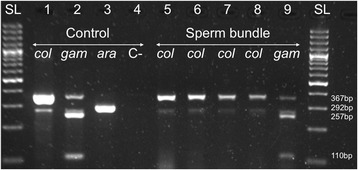
Species characterization of the sperm bundle extracted from spermathecae of inseminated females of *An. gambiae* s.l. The sperm was genotyped using a well-established PCR-RFLP species diagnostic (Fanello 2002). The lanes were: DNA size ladder (SL); positive controls for *An. coluzzii* (*col*, 1), *An. gambiae* s.s. (*gam*, 2) and *An. arabiensis* (*ara*, 3); negative control (C-, 4); sperm bundles from the spermatheca of *An. gambiae s.s.* (*col*, 5–8) and *An. coluzzii* (*gam*, 9) females from interspecific mating pairs. Expected sizes are indicated in base-pairs (bp)

In VK7, 2289 male mosquitoes sampled from 44 swarms were genotyped to species level. Of these, 41 swarms (93.18 %) were exclusively composed of *An. coluzzii*; the 3 remaining swarms (6.82 %) were a mix of *An. coluzzii* and *An. gambiae* s.s. males with a large majority of *An. coluzzii* (98.08 %, 256/261). Monospecific *An. gambiae* s.s. could not be found in VK7 (Table 1). Thirty-one free flying unpaired females collected from different swarms were identified as *An. coluzzii*. A total of 138 pairs in copula were collected from 12 swarms. Of these, 133 (96.38 %) were *An. coluzzii* pairs. The 5 remaining pairs collected in 3 swarms were heterospecific (3.62 %); 4 *An. gambiae* s.s. females paired with *An. coluzzii* males and one *An. coluzzii* female paired with an *An. gambiae* s.s. male. Monospecific *An. gambiae* mating pairs were not observed (Table 1). Repeated PCR-RFLP analyses of the sperm DNA extracted from the spermathecae showed that these interspecific mating resulted in effective sperm transfer in all 5 cases (Fig. 2). Strikingly, the rate of interspecific mating for the rare *An. gambiae* s.s. was therefore 100 % (5/5) (95 % CIs: 56.6–100) whilst for the dominant *An. coluzzii* the cross-mating rate was much lower at 3.62 % (5/138) (95 % CIs: 1.56–8.20) (Fisher-Irwin test: *N* = 143, df = 1, *P* < 0.001).

**Table 1:** Incomplete assortative mating between *An. coluzzii* and *An. gambiae* s.s. in mating swarms collected in the villages of VK7, Soumousso and Bana in Western Burkina Faso

	Swarms		Unpaired males		Unpaired females		Intraspecific pairs^a^		Interspecific pairs^a^	
Location	Swarms	Species^b^	*coluzzii*	*gambiae*	*coluzzii*	*gambiae*	*coluzzii*	*gambiae*	*coluzzii*	*gambiae*
VK7	41	*An. coluzzii*	2028	0	29	0	97	0	0	3
VK7	0	*An. gambiae*	-	-	-	-	-	-	-	-
VK7	3	mixed	256	5	2	0	36	0	1	1
Soumousso	1	*An. coluzzii*	32	0	0	0	0	0	0	0
Soumousso	27	*An. gambiae*	0	1014	0	2	0	49	0	0
Soumousso	0	mixed	-	-	-	-	-	-	-	-
Bana	18	*An. coluzzii*	120	0	0	0	-	-	-	-
Bana	19	*An. gambiae*	0	232	0	0	-	-	-	-
Bana	0	mixed	-	-	-	-	-	-	-	-
All populations	60	*An. coluzzii*	2180	0	29	0	97	0	0	3
All populations	46	*An. gambiae*	-	1246	0	2	0	49	0	0
All populations	3	mixed	256	5	2	0	36	0	1	1
Grand Total	109		2436	1251	33	0	133	49	1	4

^a^The species of females caught in intraspecific or interspecific mating pairs (in copula) is indicated

^b^Swarms are described in terms of their male species composition

No mixed swarms were detected in Soumousso and Bana (Table 1). In the former, 1014 (96.94 %) *An. gambiae* s.s. males were captured from 27 monospecific swarms and 32 (3.06 %) *An. coluzzii* males from a single monospecific swarm. Furthermore, 49 mating pairs and 2 unpaired females were collected in 13 swarms and were all identified as *An. gambiae* s.s. (Table 1). In Bana, a total of 352 males were collected from 37 monospecific swarms, with 120 (34.09 %) *An. coluzzii* males from 18 swarms and 232 (65.91 %) *An. gambiae* s.s. males from 19 swarms (Table 1).

### Female larvae and adult indoor resting females

Larval sampling was conducted in VK7 and Soumousso from July to November 2011 for time periods matching those of swarm sampling. This resulted in sexing and genotyping of 1438 wild larvae captured in 12 larval breeding sites from VK7 (36.4 %) and 21 from Soumousso (63.6 %). Among 779 larvae from Soumousso, 407 (52.2 %) were identified as females and 372 (47.8 %) as males. In VK7, the 659 larvae identified were composed of 312 (47.3 %) females and 347 (52.7 %) males. Only females were used for comparisons of relative abundances of the sibling species and detection of hybrids (Table 2).

**Table 2:** Frequencies of female *An. coluzzii*, *An. gambiae* s.s. and hybrids at the larval and adult indoor resting stages in VK7 and Soumousso during the rainy season

Locality	Life stage	Sample sizes (%)				Percent hybrization (CIs)		
		*coluzzii*	*gambiae*	Hybrids	Total	*coluzzii*	*gambiae*	Both
VK7	larvae	308 (98.72)	3 (0.96)	1 (0.32)	312	0.32 (0.05–1.81)	25.0 (4.56–69.94)	0.32 (0.06–1.81)
VK7	adults	325 (98.78)	2 (0.61)	2 (0.61)	329	0.61 (0.16–2.22)	50.0 (15.00–84.99)	0.61 (0.17–2.22)
VK7	Total	633 (98.75)	5 (0.78)	3 (0.47)	641	0.47 (0.16–1.38)	37.5 (13.68–69.42)	0.47 (0.16–1.37)
Soumousso	larvae	69 (16.95)	336 (82.56)	2 (0.49)	407	2.82 (0.78–9.70)	0.59 (0.16–2.13)	0.49 (0.13–1.78)
Soumousso	adults	108 (26.73)	295 (73.02)	1 (0.25)	404	0.92 (0.16–5.01)	0.34 (0.06–1.89)	0.25 (0.04–1.14)
Soumousso	Total	177 (21.82)	631 (77.81)	3 (0.37)	811	1.67 (0.57–4.78)	0.47 (0.16–1.38)	0.37 (0.13–1.08)
All	larvae	377 (52.43)	339 (47.15)	3 (0.42)	719	0.79 (0.27–2.30)	0.88 (0.30–0.25)	0.42 (0.14–1.22)
All	adults	433 (59.07)	297 (40.51)	3 (0.41)	733	0.69 (0.23–2.00)	1.00 (0.34–2.90)	0.41 (0.14–1.20)
All	Grand total	810 (55.78)	636 (43.80)	6 (0.41)	1452	0.74 (0.34–1.59)	0.94 (0.43–0.20)	0.41 (0.19–0.90)

Predictably, the vast majority of the 312 1-4^th^ instars larvae collected in VK7 were *An. coluzzii* (98.72 %). Only 3 *An. gambiae* s.s. (0.96 %) and one hybrid larva (0.32 %) were found. In Soumousso, 82.56 % of the 407 larvae were *An. gambiae* s.s., 16.95 % *An. coluzzii*, and 2 were hybrid larvae (0.49 %). The resulting hybridization rates at the larval stage were 0.32 and 25 % for *An. coluzzii* and *An. gambiae* s.s. in VK7 and, respectively, 2.82 and 0.59 % in Soumousso (Table 2).

A total of 733 adult indoor resting females, 329 (44.9 %) in VK7 and 404 (55.1 %) in Soumousso were collected from houses and huts for comparable time periods as the larvae and swarms sampling. The relative abundance of females followed the same patterns observed at the larval stage. In VK7, 98.78 % of adult females were *An. coluzzi*, 2 (0.61 %) *An. gambiae* s.s. and 2 (0.61 %) were hybrids. In Soumousso, 73.02 % were *An. gambiae* s.s. and 26.73 % *An. coluzzii*, whilst a single hybrid female (0.25 %) was detected. Thus, the resulting hybridization rates at the adult stage for *An. coluzzii* and *An. gambiae* s.s. were 0.61 % and 50.00 % in VK7 and 0.92 % and 0.34 % in Soumousso, respectively (Table 2).

### Hybridization rates and selection against hybrids

**Fig. 3:**
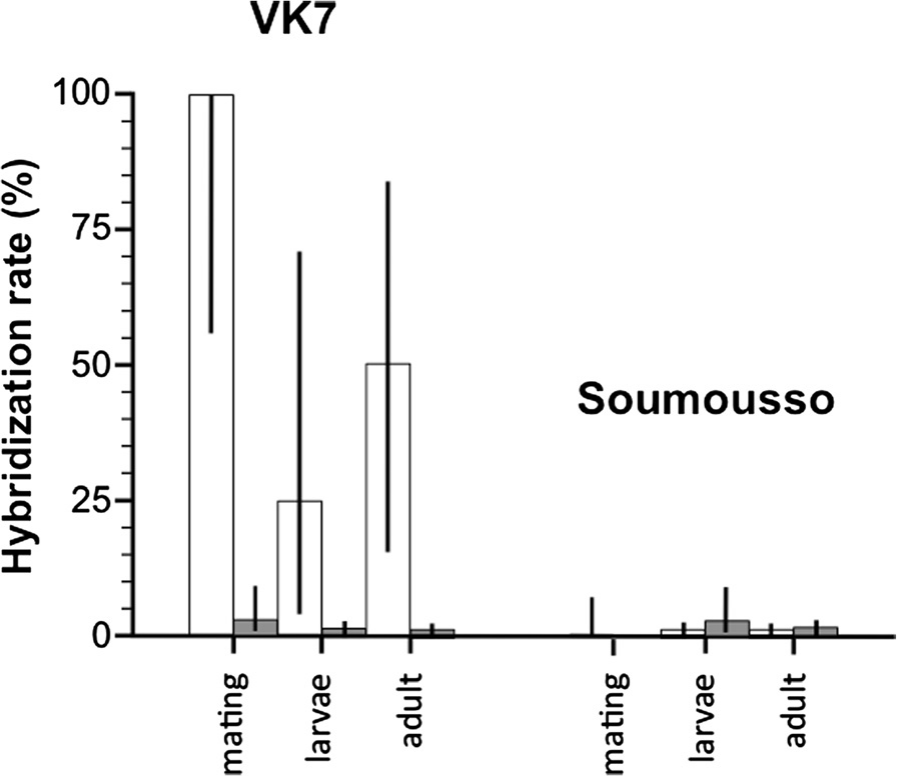
Comparison of hybridization rates at the mating stage (pairs in copula in swarms), as larvae and indoor resting stages. *An. coluzzii* and An. *gambiae s.s.* populations were sampled during the 2011 rainy season in the village of VK7 (Vallée du Kou) where *An. coluzzii* was highly dominant and in Soumousso, where *An. gambiae* s.s. dominated the malaria vectors community

Differences in hybridization rates at the mating stage (proportion mixed-species pairs), the female larval and adult stages (proportion hybrids) in the VK7 and Soumousso populations, as well as possible selection acting against hybrids from the mating to larval and to adult stage were analyzed using Logistic Regression modelling (Table 3). Hybridization rates were significantly higher in *An. gambiae* s.s, and in VK7 than in Soumousso and there was a strong interaction between species and population on hybridization rates (Fig. 3). In other words, *An. gambiae* s.s. hybridized at a very high rate when rare in VK7 but at comparable rates to *An. coluzzii* in Soumousso (Table 3). Based on the same statistical model, there was no evidence that hybrids survived less than homozygous individuals from either sibling species from the larval to the adult stage (Likelihood-ratio test on odds ratios: *P* = 0.851). However, there was a stark decrease in hybridization rates from the mating stage to the larval or adult stages (Likelihood-ratio tests on odds ratios: *P* < 0.003 in both cases) suggesting the presence of post-mating selection pressures acting shortly after cross-mating or at the early larval stages. The same results were obtained across populations when conducting separate Logistic Regressions for the rare *An. gambiae* s.s. (Likelihood-ratio tests on odd ratios: *P* < 0.002 in both cases) and the more abundant *An. coluzzii* (Likelihood-ratio tests on odds ratios: *P* < 0.0323 in all cases). Overall pair-wise differences in hybridization rates between the mating stage and larval or adult stage were also tested using Fisher-Irwin exact tests, which confirmed the results obtained by maximum likelihood (*P* < 0.034 in all cases).

**Table 3:** Logistic regression of the effects of location (VK7 and Soumousso), sibling species, and life stage on hybridization rates between *An. coluzzii* and *An. gambiae* s.s

Source	Nparm	DF	L	*P*-value
Location	1	1	9.04	0.0026
Species	1	1	16.94	<0.001
Life stage	2	2	10.78	0.005
Species*Location	1	1	27.46	<0.001

## Discussion

The results of this study confirm the importance of spatial swarm segregation as a strong reproductive isolation mechanism between *An. coluzzii* and *An. gambiae* s.s. that drastically decrease the likelihood of natural hybridization the sibling species in different locations in the Soudanian and Sahelian ecological zones [14, 37, 38]. However, this study offers new insights on the environmental conditions that promote residual hybridization and potential introgression between the cryptic taxa in their natural communities. During the rainy season of 2011, mixed swarms were only detected in the village of VK7 where *An. coluzzii* was over-abundant and *An. gambiae* s.s. extremely rare. Furthermore, no *An. gambiae* s.s. swarms were detected despite the fact that the distribution of swarms in VK7 has been extensively studied in the past decade by highly skilled swarm observers [37, 46]. Here, the few *An. gambiae* s.s. adults captured in VK7 were ‘lost’ in heterospecific swarms suggesting that they may have been unable to find conspecific mates in a habitat saturated by *An. coluzzii* swarms. The asymmetry in dominance was less pronounced in Soumousso, where no mixed swarms were detected despite the fact that rare mixed swarms were sometimes detected in previous years in that locality [13, 37, 38]. Finally in Bana, where the sibling species were found to co-exist in comparable frequencies no mixed swarms were detected either.

Crucially, the presence of rare *An. gambiae* s.s. individuals in *An. coluzzii* swarms resulted in mixed-species pairs in copula and cross-insemination as described in a previous field study in Mali [10]. At present, we do not know why cross-insemination was not confirmed in interspecific pairs captured in copula at the same Burkinabe locations in a previous study [38]. However, the technique used here allowed for re-running the PCR diagnostic several times. In addition, contamination of sperm bundles or PCR reactions by rare heterospecific DNA is less likely than contamination from maternal DNA [44], giving us confidence in the results.

Interestingly, 4 out of 5 interspecific mating involved *An. gambiae* s.s. females. This data therefore adds support to previous observations from sperm analyses [10] and inferences made from genetic analyses of adult male hybrid specimens [5], suggesting that *An. gambiae* s.s. females are more likely to cross-mate than *An. coluzzii* ones. However, because all the data suggesting higher permissiveness in *An. gambiae* s.s. females comes from rice-field areas and/or seasonal populations in which *An. gambiae* s.s. is more likely to be the rare species, understanding whether intrinsic behavioural differences between species could contribute to asymmetrical permissiveness is currently not possible.

Despite the small sample sizes, the data reported in this study suggest that females are more likely to successfully mate then males when conspecific mates are unavailable, reinforcing the idea that females drive sexual selection in anopheline swarms [8]. It is noteworthy that the adaptive value of this best-of-a-bad-job cross-mating strategy depends on the fitness costs associated with hybridization, which are thought to occur in the form of selection against *F*_1_ and *F*_1+n_ progeny resulting from these cross-inseminations. The possibility that cross-mating events occur simply as maladaptive errors, resulting in no fitness benefits, goes against increasing evidence that *F*_1_ and *F*_1+n_ individuals can be found in many populations provided adequate sampling and genotyping efforts are made [5].

Assortative mating and extrinsic barriers to reproduction in the form of selection against hybrids between *An. coluzzii* and *An. gambiae* s.s. are key to a process of incipient speciation that most assume will eventually result in intrinsic post-mating reproduction similar to those observed amongst the more diverged sibling species of the complex [6, 47, 48]. Albeit there is evidence of divergent ecological adaptations in larval habitat preferences [11, 49, 50], predation avoidance [25, 51], and adult aestivation strategy [28], decreased hybrid fitness has currently only been inferred indirectly from population genomic data [5, 19, 24, 32]. Here through extensive sampling, we show a strong correlation between hybridization rates inferred from cross-insemination rates at the mating stage and those estimated from collections of female larvae and indoor resting females performed in the same villages and spanning the same time period. Hybridization rates, strongly depended on the location considered, being very high in *An. gambiae* s.s. in VK7 where *An. coluzzii* was extremely dominant, but much lower in Soumousso were *An. gambiae* dominated. Contrary to the conclusions of a previous study [38], these data suggest that cross-mating does translate into hybrid progeny thereby creating opportunities for effective introgression between the sibling species. Hybridization rates were higher for *An. gambiae* s.s. than in *An. coluzzii* in VK7 due to its rarity, but not across both habitats. Crucially, hybridization rates significantly decreased from the mating stage to the larval stage, but not from the larval to adult stages. 

This was true for both species suggesting that selection against hybridization could occur perhaps via re-mating behaviour as suggested by patterns of cross and multiple inseminations in a previous study [52], and/or at the egg or young larval stage as suggested by evidence of divergent adaptation at the larval stage (see above).

## Conclusion

At present we do not know whether flares of hybridization, comparable to the one described here for VK7 in 2011, do take place every year in similar habitats. Previous studies at this location have repeatedly reported mixed swarms at low frequencies, suggesting that hybridization could be more common than previously thought [37, 38]. It is noteworthy that the design of the study did not allow for the detection of selection against males nor of other possible sources of selection against hybrids acting later in their life time, such as the adult mating stage or during aestivation, that could further reduce introgression. However, short of tracking the survival of individual *F*_1_ and *F*_1+n_ hybrids in the field directly, this data provides the first evidence for selection against hybrids based on field collections at different life-stages, thus supporting the idea that it plays an important role in enabling genetic divergence of the sibling species despite residual gene flow.
